# The “Hallett Sign” of Functional Jerky Movement Disorder

**DOI:** 10.1002/mds.70213

**Published:** 2026-02-23

**Authors:** Jon Stone, Anthony E. Lang, Joseph Jankovic, Michele Tinazzi, Barbara A. Dworetzky, Alan Carson, Marina A.J. Tijssen

**Affiliations:** ^1^ Centre for Clinical Brain Sciences University of Edinburgh Edinburgh UK; ^2^ Edmond J. Safra Program in Parkinson's Disease, Morton and Gloria Shulman Movement Disorders Clinic Toronto Western Hospital Toronto Ontario Canada; ^3^ Division of Neurology University of Toronto Toronto Ontario Canada; ^4^ Krembil Brain Institute Toronto Ontario Canada; ^5^ Parkinson's Disease Center and Movement Disorders Clinic, Department of Neurology Baylor College of Medicine Houston Texas USA; ^6^ Neurology Unit, Movement Disorders Division, Department of Neurosciences, Biomedicine and Movement Sciences University of Verona Verona Italy; ^7^ Department of Neurology Brigham and Women's Hospital Boston Massachusetts USA; ^8^ Expertise Centre Movement Disorders Groningen Department of Neurology University of Groningen, University Medical Centre Groningen (UMCG) Groningen The Netherlands

Mark Hallett (1943–2025), a giant in the fields of movement disorders and functional neurological disorder (FND), will be sadly missed by a global research community. We wish to note a physical sign of functional movement disorder, which we believe Mark first described and which we propose be named the “Hallett sign.”

Mark noted in 2010 that some individuals with a jerky or hyperkinetic functional movement disorder have an anticipatory jerk in expectation of tendon reflex testing, even when the hammer does not make contact with their limb.

*“Another clue that reflex myoclonus would be psychogenic is when the reflex occurs even though the tendon hammer is stopped just short of the tendon (without hitting it) – just the visual stimulus might provoke the psychogenic movement*.*”* ^1^

Mark published a video of himself (“Myoclonus 12”) (Video 1) carrying out this maneuver as part of a multiauthor book^2^ and conference on functional movement disorder held in 2009, which he often used when teaching.

**Video 1:** Mark Hallett demonstrating anticipatory jerky movements in an individual with a functional movement disorder. Reproduced with permission from Hallett et al.^2^

Anticipatory jerks like this have not been formally studied, but are identical to other stimulus‐sensitive jerky forms of functional movement disorder, originally called psychogenic myoclonus.^3^ They typically have a long and variable latency (as compared with a short latency in cortical and other “non‐functional” myoclonus), as well as a long duration of muscle contraction.^4^ These are features also shared by “voluntary” jerky movements.

The Hallett sign embodies questions surrounding free will and volition, as well as the importance of clinical diagnosis, which marked Mark Hallett's career in neurophysiology, movement disorders, and as the first president of the FND Society.

## Author Roles

(1) Research Project: A. Conception, B. Organization, C. Execution; (2) Statistical Analysis: A. Design, B. Execution, C. Review and Critique; (3) Manuscript: A. Writing of the First Draft, B. Review and Critique.

J.S.: 1A, 3A, 3B.

A.E.L.: 3B.

J.J.: 3B.

M.T.: 3B.

B.D.: 3B.

A.C.: 3B.

M.A.J.T.: 3B.

## Financial Disclosures and Conflicts of Interest

Author disclosures are available in the Supporting Information.

## Supporting information


**Data S1.** Coi_Disclosure.
